# Late Gadolinium Enhancement Predicts Improvement in Global Longitudinal Strain after Aortic Valve Replacement in Aortic Stenosis

**DOI:** 10.1038/s41598-019-51930-2

**Published:** 2019-10-30

**Authors:** Tsuyoshi Fujimiya, Masumi Iwai-Takano, Takashi Igarashi, Hiroharu Shinjo, Keiichi Ishida, Shinya Takase, Hitoshi Yokoyama

**Affiliations:** Department of Cardiovascular Surgery, Fukushima Medical University, Fukushima, Japan

**Keywords:** Cardiology, Valvular disease

## Abstract

Myocardial fibrosis, as detected by late gadolinium enhancement (LGE) magnetic resonance imaging (MRI), is related to mortality after aortic valve replacement (AVR). This study aimed to determine whether LGEMRI predicts improvement in global longitudinal strain (GLS) after AVR in patients with severe aortic stenosis (AS). Twenty-nine patients with severe AS who were scheduled to undergo AVR were enrolled. Two-dimensional echocardiography and contrast-enhanced MRI were performed before AVR. GLS and LGEcore (g: > 5 SD of normal area), LGEgray (g: 2–5 SD), and LGEcore+gray (g) were measured. One year after AVR, GLS were examined by echocardiography to assess improvement in LV function. Preoperatively, GLS correlated with LGEcore (g) (r^2^ = 0.14, p < 0.05), LGEgray (g) (r^2^ = 0.32, p < 0.01) and LGEcore+gray (g) (r^2^ = 0.36, p < 0.01). LGEcore was significantly lower in patients with improved GLS after AVR (GLS_1year_ ≥ −19.9%) compared to those with no improvement (1.34 g vs. 4.70 g, p < 0.01). LGE predicts improvement in LV systolic function after AVR.

## Introduction

Aortic stenosis (AS) remains therapeutic challenge especially in elderly patients. Left ventricular (LV) myocardial fibrosis is associated with progression of LV hypertrophy, which compensates for pressure overload in patients with AS. Myocardial fibrosis is classified as focal fibrosis or diffuse fibrosis, with the latter being an early phenomenon preceding the former^1^. LV myocardial advanced fibrosis, especially focal fibrosis or scars, reportedly correlates with LV systolic dysfunction, and the severity of fibrosis is known to be associated with a poor late prognosis^2^. In some cases, LV dysfunction and heart failure further progress after aortic valve replacement (AVR). Therefore, the optimal timing for AVR needs to be determined while considering the grade of LV myocardial fibrosis. While myocardial biopsy is the gold standard for detecting myocardial fibrosis, its general applicability is limited due to the invasiveness of the procedure.

Cardiac magnetic resonance imaging (MRI) is widely used for assessment of myocardial fibrosis^1^. Late gadolinium enhancement (LGE) MRI is a useful method for detecting focal myocardial fibrosis^1^. Myocardial fibrosis detected by LGE has been reported to correlate with mortality in patients with AS during a median follow up of 2.9 years after AVR^3^.

Several studies have reported that global longitudinal strain (GLS), an index of LV systolic function assessed by echocardiography, is reduced even in AS patients with preserved LV ejection fraction (EF)^4^. Impaired GLS is known to correlate with AS severity, increased left ventricular mass index (LVMI)^5^, and all-cause mortality in patients with AS^6^. However, few studies have examined which preoperative examinations predict improvement in GLS after AVR.

This study aimed to examine whether LGE MRI predicts improvement in GLS after AVR in patients with severe AS.

## Results

### Baseline characteristics

Table 1 summarized preoperative baseline characteristics of the 29 patients (age, 73 years; 52% male) included in this study. Nine patients presented with symptoms of heart failure, and 16 patients presented with symptoms of AS. In this cohort, patients had several atherosclerotic risk factors (hypertension, diabetes mellitus, hyperlipidemia, and/or current smoking).

**Table 1:** Patient baseline characteristics (n = 29).

Age, yrs	73 (66–78)
Men, n (%)	15 (52)
Height, cm	155.4 (148.2–161.7)
Body weight, kg	55.2 (51.8–60.5)
Body surface area, m^2^	1.50 (1.41–1.60)
Body mass index, kg/m^2^	22.8 (19.8–25.0)
**NYHA functional class**, **n (%)**	
I	20 (69)
II	8 (28)
III	1 (3)
IV	0
**Symptoms, n (%)**	
Dyspnea	9 (31)
Chest pain	4 (14)
Syncope	3 (10)
**Risk factors, n (%)**	
Hypertension	19 (66)
Diabetes mellitus	4 (14)
Hyperlipidemia	16 (55)
Current smoker	5 (17)
**History/comorbidity, n (%)**	
Chronic kidney disease	7 (24)
Cerebral vascular disease	3 (10)
Chronic obstructive pulmonary disease	3 (10)
**Medication, n (%)**	
β blocker	5 (17)
ACE inhibitor	2 (7)
ARB	15 (52)
Anti-aldosterone	2 (7)
Diuretics	2 (7)
Brain natriuretic peptide, pg/ml	85.0 (39.1–183.0)
eGFR, ml/min/1.73 m^2^	68.0 (59.0–74.0)
Logistic Euro score, %	5.13 (2.54–6.41)

Continuous variables are expressed as median (interquartile range).

NYHA: New York Heart Association, ACE: angiotensin converting enzyme, ARB: angiotensin II receptor blocker, GFR: estimated glomerular filtration rate.

Table 2 shows echocardiographic parameters at baseline. All patients had high-gradient severe AS. LVEF was well-preserved at 65.7%, while GLS was reduced at −16.5%. LV hypertrophy was observed, but no severe diastolic dysfunction with increased LA pressure was noted.

**Table 2:** Preoperative echocardiographic and MRI parameters (n = 29).

**Echocardiography**	
IVS, mm	13.1 (11.1–14.3)
PW, mm	12.9 (11.4–13.5)
LVDd, mm	41.7 (37.4–45.5)
LVDs, mm	25.4 (21.9–28.7)
LVEDV, ml	62.8 (54.1–77.1)
LVESV, ml	21.5 (17.2–28.7)
LV ejection fraction, %	65.7 (61.9–68.5)
LVMI, g/m^2^	123.2 (113.0–148.6)
LAVI, ml/m^2^	37.4 (25.7–48.8)
E/A	0.63 (0.51–0.82)
e′, cm/sec	4.9 (4.4–6.2)
E/e′	12.4 (9.4–19.5)
Peak velocity, m/s	4.72 (4.30–5.25)
Mean PG, mmHg	51.0 (41.4–68.1)
Aortic valve area, cm^2^	0.67 (0.57–0.79)
Zva, mmHg/ml/m^2^	5.40 (4.53–6.50)
2D-GLS, %	−16.5 (−18.2–14.2)
**MRI**	
LGEcore, g	3.0 (1.2–6.7)
LGEcore, %	3.9 (1.2–8.4)
LGEcore, g/m^2^	2.5 (0.7–5.8)
LGEgray, g	10.8 (7.3–17.8)
LGEgray, %	11.4 (8.0–20.9)
LGEgray, g/m^2^	8.0 (4.6–13.0)
LGEcore+gray, g	15.0 (9.5–22.7)
LGEcore+gray, %	14.3 (10.9–28.5)
LGEcore+gray, g/m^2^	10.6 (5.9–17.7)

Continuous variables are expressed as median (interquartile range).

IVS: interventricular septal thickness, PW: posterior wall thickness, LVDd: left ventricular end-diastolic diameter, LVDs: left ventricular end-systolic diameter, LVEDV: left ventricular end-diastolic volume, LVESV: left ventricular end-systolic volume, LVMI: left ventricular mass index, LAVI: left atrium volume index, PG: pressure gradient, GLS: global longitudinal strain, LGE: late gadolinium enhancement, BSA: body surface area.

The parameters of myocardial fibrosis by MRI were showed no severe myocardial fibrosis. As shown in Fig. 2A, significant correlations were observed between GLS and LGEcore (g) (r^2^ = 0.14, p < 0.05), LGEgray (g) (r^2^ = 0.32, p < 0.01) and LGEcore+gray (g) (r^2^ = 0.36, p < 0.01).

**Figure 1:**
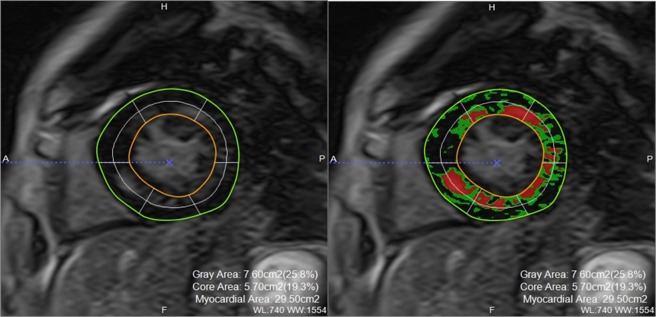
Measurement of LGE by MRI. LGEcore, LGEgray, and LGEcore+gray were calculated as areas with the above-threshold signal intensity in the ROI (≥5 SD for LGEcore and 2–5 SD for LGEgray compared to the normal area). ROI: region of interest. LGE: late gadolinium enhancement.

**Figure 2:**
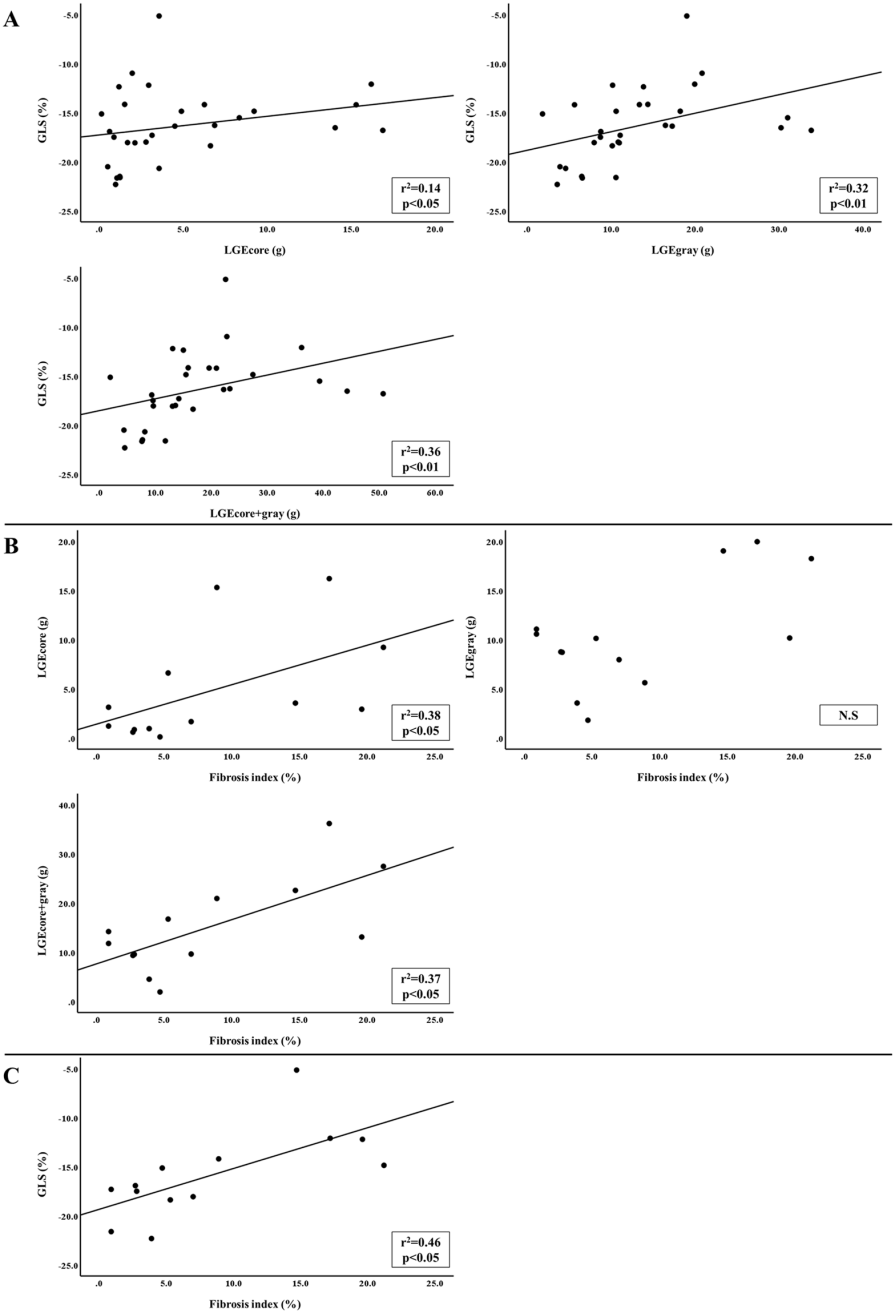
Correlation between GLS, LGE and FI. (**A**) GLS was significantly correlated with LGEcore (g) (r^2^ = 0.14, p < 0.05), LGEgray (g) (r^2^ = 0.32, p < 0.01) and LGEcore+gray (g) (r^2^ = 0.36, p < 0.01). (**B**) LGEcore (g) (r^2^ = 0.38, p < 0.05) and LGEcore+gray (g) (r^2^ = 0.37, p < 0.05), but not LGEgray, was significantly correlated with the fibrosis index. (**C**) GLS was significantly correlated with the fibrosis index (r^2^ = 0.46, p < 0.05).

### Relationships between myocardial fibrosis and imaging parameters

The fibrosis index (FI) obtained from myocardial biopsy specimens of 13 patients was 5.3% (interquartile range [IQR] 2.8–16.0). FI correlated with LGEcore (g) (r^2^ = 0.38, p < 0.05) and LGEcore+gray (g) (r^2^ = 0.37, p < 0.05), but not LGEgray (g) (Fig. 2B). FI strongly correlated with GLS (r^2^ = 0.46, p < 0.05) (Fig. 2C).

### Follow-up echocardiography after AVR

There was no all-cause death or hospitalization due to heart failure at one year after AVR. We examined echocardiography in 26 patients; reasons for not performing follow-up echocardiography included patient refusal, cost of echocardiography, and other socioeconomical reasons. The results of comparisons of echocardiographic parameters before and after AVR are summarized in Table 3. After AVR, aortic valve function was significantly improved in terms of peak velocity (4.73 to 2.55 m/s), mean pressure gradient (50.5 to 14.1 mmHg), aortic valve area (0.65 to 1.47 cm^2^), and valvulo-arterial impedance (Zva) (5.46 to 4.48 mmHg/ml/m^2^). There were no paravalvular leakage. Regression of LV hypertrophy and a significant improvement in GLS were observed after AVR (−16.9% to −19.9%).

**Table 3:** Comparison of pre- and postoperative echocardiographic parameters (n = 26).

	Pre-AVR	Post-AVR	P value
IVS, mm	13.1 (11.5–14.2)	10.8 (9.0–12.2)	<0.001
PW, mm	13.0 (11.6–13.4)	10.1 (9.0–11.6)	<0.001
LVDd, mm	41.5 (37.3–46.3)	43.0 (37.4–45.2)	0.76
LVDs, mm	25.6 (22.0–29.0)	25.7 (21.0–29.6)	0.80
LVEDV, ml	62.9 (54.4–78.7)	63.6 (56.3–79.7)	0.88
LVESV, ml	21.9 (17.3–31.2)	23.2 (19.5–30.2)	0.74
LV ejection fraction, %	65.6 (61.8–68.7)	65.6 (57.6–67.4)	0.34
LVMI, g/m^2^	123.2 (113.1–142.3)	92.9 (81.1–110.0)	<0.001
LAVI, ml/m^2^	35.5 (20.8–48.7)	30.6 (24.4–39.0)	0.28
E/A	0.62 (0.51–0.82)	0.91 (0.73–1.14)	0.014
e’, cm/sec	5.2 (4.5–6.4)	7.5 (5.9–9.6)	0.001
E/e’	12.1 (8.8–14.5)	9.7 (8.1–14.2)	0.38
Peak velocity, m/s	4.73 (4.18–5.35)	2.55 (2.44–3.01)	<0.001
Mean PG, mmHg	50.5 (39.4–70.0)	14.1 (11.5–17.1)	<0.001
Aortic valve area, cm^2^	0.65 (0.56–0.76)	1.47 (1.20–1.75)	<0.001
Zva, mmHg/ml/m^2^	5.46 (4.98–6.51)	4.48 (3.37–5.04)	0.001
2D-GLS, %	−16.9 (−18.9–14.2)	−19.9 (−22.1–17.9)	0.004

Continuous variables are expressed as median (interquartile range).

IVS: interventricular septal thickness, PW: posterior wall thickness, LVDd: left ventricular end-diastolic diameter, LVDs: left ventricular end-systolic diameter, LVEDV: left ventricular end-diastolic volume, LVESV: left ventricular end-systolic volume, LVMI: left ventricular mass index, LAVI: left atrium volume index, PG: pressure gradient, GLS: global longitudinal strain.

We divided the 26 patients who underwent follow-up echocardiography according to median postoperative GLS: the improvement group (≥−19.9%; n = 14) and the non-improvement group (<−19.9%; n = 12). The comparisons of patient characteristics, echocardiographic parameters, and MRI parameters between the two groups are shown in Table 4.

**Table 4:** Comparison of echocardiographic and MRI parameters between groups with or without GLS improvement.

	Improvement group (n = 14)	Non-improvement group (n = 12)	P value
Age, yrs	73.0 (65.5–78.3)	72.5 (58.8–75.8)	0.71
Hypertension, n (%)	9 (64)	8 (67)	0.90
Diabetes, n (%)	2 (14)	2 (17)	0.87
**Medication**			
β blocker, n (%)	1 (7)	3 (25)	0.21
ACE inhibitor, n (%)	1 (7)	1 (8)	0.91
ARB, n (%)	6 (43)	6 (50)	0.72
Anti-aldosterone, n (%)	0	1 (8)	0.27
Diuretics, n (%)	0	1 (8)	0.27
**Implanted valve size**			0.26
19 mm, n	7	4	
21 mm, n	5	7	
23 mm, n	2	0	
27 mm, n	0	1	
Preoperative sBP, mmHg	117 (102–136)	121 (117–131)	0.71
Postoperative sBP, mmHg	126 (118–136)	131 (114–140)	0.56
**Preoperative echocardiography**			
IVS, mm	13.2 (12.1–14.7)	13.0 (10.5–14.0)	0.63
PW, mm	12.8 (11.6–13.6)	13.1 (11.5–13.5)	0.71
LVDd, mm	40.9 (37.5–42.2)	45.5 (37.0–50.3)	0.13
LVDs, mm	24.4 (19.5–27.7)	26.1 (22.8–32.0)	0.19
LVEDV, ml	60.8 (51.7–70.4)	69.5 (57.6–90.2)	0.11
LVESV, ml	19.2 (16.0–26.5)	25.3 (17.6–31.7)	0.25
LV ejection fraction, %	66.0 (60.5–70.1)	65.4 (62.4–67.2)	0.90
LVMI, g/m^2^	118.9 (108.7–137.8)	127.7 (115.2–164.6)	0.37
LAVI, ml/m^2^	37.4 (20.8–48.7)	33.5 (22.2–58.6)	0.98
E/A	0.62 (0.51–0.71)	0.68 (0.51–1.18)	0.35
e’, cm/sec	5.3 (4.5–6.2)	5.2 (4.5–7.0)	0.94
E/e’	10.6 (8.4–14.5)	12.6 (10.1–18.2)	0.32
Peak velocity, m/s	4.73 (4.18–5.38)	4.75 (4.07–5.43)	0.98
Mean PG, mmHg	52.0 (40.8–76.8)	50.5 (38.1–68.6)	0.78
Aortic valve area, cm^2^	0.64 (0.54–0.77)	0.66 (0.56–0.81)	0.67
SVi, ml/m^2^	34.5 (25.6–35.6)	31.4 (24.6–36.0)	0.61
Zva, mmHg/ml/m^2^	5.81 (4.10–7.07)	5.41 (4.99–6.27)	0.76
2D-GLS, %	−17.7 (−20.5–14.9)	−15.2 (−18.1–12.7)	0.18
**Postoperative echocardiography**			
IVS, mm	9.7 (8.7–11.8)^*^	11.5 (9.9–13.4)	0.041
PW, mm	9.5 (8.6–10.6)^*^	11.1 (9.9–13.0)	0.036
LVDd, mm	43.0 (38.0–44.4)	41.8 (37.4–45.4)	0.86
LVDs, mm	24.9 (21.4–29.6)	25.7 (20.1–29.7)	0.82
LVEDV, ml	62.9 (55.1–79.7)	65.3 (55.9–81.9)	0.63
LVESV, ml	24.8 (17.6–30.6)	22.1 (20.4–29.2)	0.94
LV ejection fraction, %	65.2 (57.5–67.4)	65.5 (61.4–68.4)	0.82
LVMI, g/m^2^	82.2 (74.5–101.2)^*^	102.7 (92.4–127.6)^*^	0.036
LAVI, ml/m^2^	29.8 (22.9–34.1)	37.2 (24.1–52.2)	0.30
E/A	0.97 (0.79–1.25)^*^	0.9 (0.6–1.1)	0.32
e’, cm/sec	8.5 (6.9–10.6)^*^	6.0 (5.1–8.0)	0.011
E/e’	9.2 (7.1–11.9)	11.6 (9.3–15.8)	0.044
Peak velocity, m/s	2.6 (2.5–3.1)^*^	2.5 (2.3–3.0)^*^	0.53
Mean PG, mmHg	15.7 (12.1–17.1)^*^	13.0 (11.2–17.7)^*^	0.49
Aortic valve area, cm^2^	1.26 (1.12–1.61)^*^	1.60 (1.32–1.79)^*^	0.28
SVi, ml/m^2^	35.1 (32.7–40.3)^†^	33.3 (24.7–40.1)	0.33
Zva, mmHg/ml/m^2^	4.11 (3.39–4.78)^*^	4.42 (3.15–5.58)	0.33
2D-GLS, %	−22.1 (−22.4–20.3)^*^	−17.6 (−18.7–13.4)	0.001
**Preoperative MRI**			
LGEcore, g	1.34 (0.81–2.98)	4.70 (2.99–9.00)	0.005
LGEcore, %	1.40 (1.10–4.43)	5.50 (2.03–8.50)	0.036
LGEcore, g/m^2^	0.91 (0.59–2.23)	3.39 (1.83–6.28)	0.015
LGEgray, g	8.72 (4.40–13.48)	10.8 (10.1–18.8)	0.12
LGEgray, %	11.0 (7.23–14.3)	10.4 (8.35–20.6)	0.46
LGEgray, g/m^2^	5.61 (3.10–8.67)	7.55 (6.13–12.7)	0.12
LGEcore+gray, g	9.62 (6.84–15.24)	18.8 (13.4–26.2)	0.013
LGEcore+gray, %	12.9 (8.53–16.1)	15.8 (12.0–26.7)	0.16
LGEcore+gray, g/m^2^	6.49 (4.50–10.5)	11.6 (8.19–18.1)	0.023

ACE: angiotensin converting enzyme, ARB: angiotensin II receptor blocker, sBP: systolic blood pressure, IVS: interventricular septal thickness, PW: posterior wall thickness, LVDd: left ventricular end-diastolic diameter, LVDs: left ventricular end-systolic diameter, LVEDV: left ventricular end-diastolic volume, LVESV: left ventricular end-systolic volume, LVMI: left ventricular mass index, LAVI: left atrium volume index, PG: pressure gradient, SVi: stroke volume index, GLS: global longitudinal strain, LGE: late gadolinium enhancement, BSA: body surface area.

^*^p < 0.01 vs. preoperative echocardiography.

^†^p < 0.05 vs. preoperative echocardiography.

No significant differences were observed in age, implanted valve size, and blood pressure between the improvement and non-improvement groups. Preoperative echocardiographic parameters did not differ between the two groups. Postoperatively, however, significant improvements were observed in LV hypertrophy (interventricular septal thickness [IVS] and posterior wall thickness [PW]) and LV diastolic function (left ventricular mass index [LVMI] and e') in the improvement group compared to the non-improvement group.

LGEcore and LGEcore+gray were lower in the improvement group compared to the non-improvement group. LGEgray did not differ between the two groups.

In the univariate analysis, LGEcore (g), LGEcore (% of LV) and LGEcore (g/BSA) were significant predictors of GLS improvement after AVR (LGEcore [g]: β = 0.446, p = 0.011; LEGcore [% of LV]: β = 0.452, p = 0.020; LGEcore [g/BSA]: β = 0.417, p = 0.034) (Table 5). On the other hand, no preoperative echocardiographic parameters including GLS, LVMI, wall thickness, diastolic indices, hypertension, diabetes and medication predicted improvement in GLS. In the multivariate analysis, LGEcore (g) was found to be an independent predictor of postoperative improvement in GLS (β = 0.446, p = 0.022) (Table 5).

**Table 5:** Multivariate analysis to predict postoperative improvement in GLS.

	Univariate analysis		Multivariate analysis	
	β	P value	β	P value
Preoperative GLS	0.264	0.10		
LGEcore (g)	0.446	0.011	0.446	0.022
LGEcore (% of LV)	0.452	0.020		
LGEcore (g/BSA)	0.417	0.034		
LGEcore+gray (g)	0.319	0.056		
LGEcore+gray (% of LV)	0.313	0.120		
LGEcore+gray (g/BSA)	0.276	0.173		

GLS: global longitudinal strain, LGE: late gadolinium enhancement, LV: left ventricle, BSA: body surface area, ACE: angiotensin converting enzyme, ARB: angiotensin II receptor blocker.

**Figure 3:**
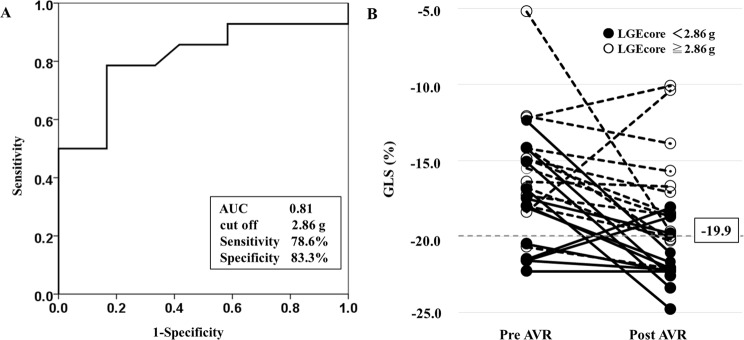
Receiver-Operating Characteristics (ROC) Curve Analysis for Prediction of GLS Improvement after AVR. (**A**) In the ROC analysis, the area under the curve was 0.81 for predicting postoperative GLS improvement (≥−19.9%) by LGEcore, with a cut-off value of 2.86 g (sensitivity, 78.6%; specificity, 83.3%). (**B**) Patients with low LGEcore (<2.86 g) showed improvement in GLS after AVR compared to those with high LGEcore (≥2.86 g). Black circle: LGEcore <2.86 g, white circle: LGEcore ≥2.86 g.

In the ROC analysis, the area under the curve was 0.81 for predicting postoperative GLS improvement (≥−19.9%) by LGEcore (g), with a cut-off value of 2.86 g (sensitivity, 78.6%; specificity, 83.3%) (Fig. 3A).

Figure 3B shows changes in GLS before and after AVR for each patient. Patients with low LGEcore (<2.86 g) showed improved GLS after AVR compared to those with high LGEcore (≥2.86 g).

## Discussion

In this study, we investigated whether preoperative LGEs could predict improvement in GLS after AVR in patients with preserved LVEF and reduced GLS. The major findings are as follows: 1) Preoperative examinations revealed significant correlations among GLS, LGEs, and FI; 2) One year after AVR, GLS was improved in a manner dependent on preoperative LGEcore; and 3) LGEcore can predict postoperative improvement in GLS with a cut-off value of 2.86 g. These findings suggest that myocardial fibrosis as detected by LGE predicts improvement in GLS after AVR.

### Relationships among GLS, LGEs, and FI

In the present study, GLS correlated with LGEcore+gray. On the other hand, the correlation with LGEcore was weak. LGEgray didn’t correlate with FI. Because interstitial diffuse fibrosis, interstitial edema and myocardial hypertrophy weren’t detected as FI, LGEgray containing them didn’t correlate with FI. GLS correlated well with LGEcore+gray including focal and diffuse fibrosis because GLS reflected the state of the whole myocardium.

Microscopic changes in LV are characterized by cardiomyocyte hypertrophy and extracellular matrix expansion in patients with AS. These conditions are caused by either focal replacement fibrosis (scar) or reactive and interstitial diffuse fibrosis^2,3,7–11^. According to previous studies, LGEcore and LGEgray reflect focal fibrosis and diffuse mild interstitial fibrosis, respectively^12^.

In a previous study, histological findings suggested improved GLS in patients with mild fibrosis^9^. Lee *et al*. also reported that native T1 values by cardiac MRI as an index of diffuse interstitial fibrosis correlated with GLS^13^. Reduction of GLS correlates with several factors such as myocardial fibrosis^14^, pressure overload, and obesity^15^, improved after AVR if fibrosis was mild in previous report^9^. Although the main cause of GLS impairment is still unknown, as well as the prospect for GLS improvement after AVR, it suggests that myocardium of patients with severe AS contains reversible and irreversible fibrosis.

GLS is reduced in symptomatic patients with severe AS, and a decrease in GLS is a predictor of all-cause mortality^6^. GLS is also a predictor of future major adverse cardiac events in asymptomatic patients with severe AS and preserved LVEF^16^. Thus, assessing GLS is clinically important in patients with potential systolic dysfunction and preserved LVEF. In the present cohort study, patients had preserved LVEF with a slight decrease in GLS, and myocardial specimens showed mild fibrosis compared to severity of fibrosis in the previous reports^13^. However, given that not all patients showed improved GLS after AVR, predictors of GLS improvement after AVR need to be investigated further.

### Prediction of improvement in GLS after AVR

In the present study, LGEcore, but not LGEgray or LGEcore+gray, was found to be a predictor of GLS improvement after AVR. While LGEgray (i.e., mild interstitial fibrosis) can be reversible, LGEcore (i.e., focal fibrosis) is unlikely to improve after AVR. Thus, our findings suggest that the degree of focal fibrosis before AVR is a determining factor for GLS improvement after AVR in patients with severe AS.

A previous cohort study reported that LGE did not improve significantly 9 months after AVR^9^. Since focal fibrosis doesn’t improve, the decrease in diffuse fibrosis contributes to the improvement of postoperative contractility. A recent prospective observational cohort study reported that focal fibrosis (scars) as detected by LGE does not resolve, while diffuse fibrosis and myocardial hypertrophy as assessed by extracellular volume (ECV) show significant regression after AVR in patients with symptomatic severe AS^7^. However, it remains unclear as to which type of LV myocardial fibrosis (i.e., focal or diffuse) plays an important role in persistent systolic dysfunction after AVR.

To the best of our knowledge, this is the first study to evaluate whether LGE as an index of focal fibrosis and/or diffuse fibrosis could predict improvement in GLS one year after AVR. Preoperative GLS strongly correlated with LGEgray, but weakly correlated with LGEcore. On the other hand, LGEcore was found to be a predictor of GLS improvement after AVR. The use of different thresholds, i.e., > 5 standard deviation (SD) for LGEcore and 2–5 SD for LGEgray, allowed us to detect potential systolic dysfunction with preserved LVEF (LGEgray), and to predict improvement in GLS (LGEcore) after AVR.

### Clinical implication

Recent therapeutic strategies for asymptomatic severe AS include AVR, which is recommended only when LVEF is less than 50%^17^. However, severe AS patients with preserved LVEF already has LV myocardial fibrosis^18^. In patients with extensive focal fibrosis, myocardial damage persists even if LV afterload is decreased by AVR. Thus, myocardial fibrosis needs to be evaluated noninvasively in order to predict prognosis after AVR in a clinical setting. Since focal fibrosis as detected by LGEcore (<2.86 g) is an independent predictor of GLS improvement after AVR, surgical therapy should be considered before patients develop irreversible LV dysfunction.

### Study limitations

This study has several limitations. First, this study was conducted at a single center. Second, we excluded patients with chronic kidney disease (CKD) because of a contraindication to contrast-enhanced MRI. Thus, the results of the present study may not apply to patients with CKD, which is a common disorder in elderly patients. Other methods to assess LV myocardial fibrosis, e.g., ECV by MRI^19,20^, should be considered. Third, we assessed GLS by 2D echocardiography, not 3D echocardiography. A significant correlation has been reported between 2D GLS and 3D GLS in patients with AS, and 3D GLS as well as 2D GLS are reportedly predictors of major adverse cardiac events^13^. Forth, the myocardial specimens were endomyocardial and not transmural which raised issue of representability.

In conclusion, this prospective observational study demonstrated that LGEcore predicts improvement in GLS after AVR in patients with severe AS and preserved LVEF.

## Methods

### Study design and patient recruitment

This prospective observational study was conducted in 29 patients with severe AS who underwent AVR from January 2014 to July 2017. Severe AS was defined as an aortic valve area < 1.0 cm^2^, peak aortic valve velocity > 4.0 m/s, and mean pressure gradient > 40 mmHg^21^. Exclusion criteria were patients with concomitant severe aortic regurgitation, moderate to severe mitral regurgitation, and a previous history of ischemic heart disease, atrial fibrillation, left bundle branch block, or CKD (eGFR < 30 ml/min/1.73 m^2^ is a contraindication to gadolinium enhanced-MRI)^22^.

All patients underwent echocardiography and MRI prior to AVR. Myocardial biopsy specimens were collected intraoperatively from 13 patients who obtained informed consent. We evaluated relationships among the parameters of echocardiography and MRI and myocardial specimens. Echocardiography was also performed one year after AVR to assess the correlation between preoperative LGE and postoperative GLS improvement.

This study was approved by the institutional review board of Fukushima Medical University and was conducted in compliance with the principles of the Declaration of Helsinki. All patients provided written informed consent.

### Echocardiography

We performed transthoracic echocardiography to assess aortic valve function and LV systolic and diastolic function using the Acuson SC2000^TM^ system (SIEMENS: Mountain View, CA, USA) with a 4-MHz transducer^23,24^. Echocardiographic parameters included LV wall thickness and dimension, LV volume and LVEF, left atrium volume index (LAVI), peak early filling (E velocity) and late filling (A velocity) using pulsed-wave Doppler images, peak early diastolic velocity (mean E´of lateral and septal wall) using tissue Doppler images, E/e' and AS indices (aortic valve area, peak velocity, mean pressure gradient [MPG], and Zva). Zva was defined as the ratio of estimated LV systolic pressure (the sum of systolic arterial pressure [SAP] and MPG) to stroke volume index (SVi): Zva = (SAP + MPG)/SVi^25^. LV mass index was calculated by the cube formula in the parasternal long-axis view^23^.

2D-GLS was examined by 2D speckle tracking echocardiography using the SC2000 workplace system VVI^TM^ (SIEMENS: Mountain View, CA, USA). We assessed endocardial GLS as the average of GLSs in apical 2-, 3-, and 4-chamber views^26^.

### Cardiac MRI

Cardiac MRI was performed on a 1.5-T scanner (Vantage Titan^TM^: Canon Medical Systems, Otawara, Japan) according to the standard LGE protocol^27^. Ten minutes before image acquisition, 1.0 M gadobutrol (Gadovist^TM^: Bayer, Berlin, Germany), a gadolinium-based contrast agent, was administered systemically to patients with eGFR ≥ 30 ml/min/1.73 m^2^.

Cardiac MRI was analyzed using a post-processing workstation (Ziostation2^TM^: Ziosoft, Tokyo, Japan). The contours of the LV endocardium and epicardium were traced semi-automatically in short-axis slices. The region of interest (ROI) was selected within the remote reference myocardium to set the SD^28^. All LGE measurements were performed by the author (T.F), and 95% intraclass correlation coefficient was 0.858. We evaluated LGEs as parameters of fibrosis, calculated on the workstation as areas with the above-threshold signal intensity compared to the remote reference myocardium in the ROI (LGEcore: > 5 SD; LGEgray: 2–5 SD; LGEcore+gray: LGEcore plus LGEgray) (Fig. 1)^12,29^. These indices were expressed as absolute amounts (g), percentage of myocardial mass (% of LV), and amounts corrected by body surface area (g/BSA).

### Intraoperative biopsy

Intraoperative myocardial biopsy specimens were taken from 13 of the 29 patients. An endomyocardial specimen roughly 8 mm^3^ in volume was obtained from the basal muscular septum 2 cm below the outflow tract following aortic valve resection. All specimens were preserved in 20% formalin, embedded in paraffin, cut into 5-µm-thick sections, and stained with Elastica-Masson stain. The myocardial muscle and fibrous tissue was observed at a magnification of 100x^30^. The FI was defined as the ratio (in percentage) of fibrosis tissue to the total myocardial field using Image J^31^. For each patient, FI was quantified in five different fields representative of all myocardial samples.

### Relationships among echocardiography, MRI, and myocardial specimens at baseline and follow-up

We evaluated relationships among preoperative GLS by echocardiography, LGEcore, LGEgray, and LGEcore+gray by MRI, and FI derived from myocardial specimens.

Patients underwent echocardiography one year after AVR and were divided into the following two groups according to GLS improvement: the improvement group (postoperative GLS greater than or equal to median) and the non-improvement group (post-operative GLS less than median).

Pre- and postoperative echocardiographic parameters and LGEs were compared between the improvement group and the non-improvement group in order to assess whether it is possible to predict improvement in GLS after AVR. Moreover, multivariate analysis was performed to determine which parameters are independent predictors of GLS improvement.

### Statistical analysis

Statistical analyses were performed using SPSS^TM^ software version 23 (IBM, Armonk, New York). Categorical variables were expressed as percentages. All continuous variables were expressed as a median (IQR). Comparisons between the improvement and non-improvement group were assessed by the Mann-Whitney U test for non-normally distributed variables and the chi-square test for categorical variables. On the other hand, the comparison of pre- and postoperative results was assessed by Wilcoxon paired t test. Log transformation was used to normalize the distribution of preoperative GLS, LGEcore, and LGEcore+gray. Multiple linear regression was used to predict postoperative improvement in GLS based on preoperative GLS, LGEcore, and LGEcore+gray. For each parameter, log-converted values were used for multivariate analysis (i.e., x: [log x]/[SD of log x]).
